# Graphene-Reinforced Titanium Enhances Soft Tissue Seal

**DOI:** 10.3389/fbioe.2021.665305

**Published:** 2021-04-13

**Authors:** Jianxu Wei, Shichong Qiao, Xiaomeng Zhang, Yuan Li, Yi Zhang, Shimin Wei, Junyu Shi, Hongchang Lai

**Affiliations:** Department of Oral and Maxillo-facial Implantology, Shanghai Ninth People’s Hospital, School of Medicine, Shanghai Jiao Tong University, Shanghai, China

**Keywords:** fibroblasts, bacteria, bioactive materials, peri-implant infections, sequencing

## Abstract

The integrity of soft tissue seal is essential for preventing peri-implant infection, mainly induced by established bacterial biofilms around dental implants. Nowadays, graphene is well-known for its potential in biocompatibility and antisepsis. Herein, a new titanium biomaterial containing graphene (Ti-0.125G) was synthesized using the spark plasma sintering (SPS) technique. After material characteristics detection, the subsequent responses of human gingival fibroblasts (HGFs) and multiple oral pathogens (including *Streptococci mutans*, *Fusobacterium nucleatum*, and *Porphyromonas gingivalis*) to the graphene-reinforced sample were assessed, respectively. Also, the dynamic change of the bacterial multispecies volume in biofilms was evaluated using absolute quantification PCR combined with Illumina high-throughput sequencing. Ti-0.125G, in addition to its particularly pronounced inhibitory effect on *Porphyromonas gingivalis* at 96 h, was broadly effective against multiple pathogens rather than just one strain. The reinforced material’s selective responses were also evaluated by a co-culture model involving HGFs and multiple strains. The results disclosed that the graphene-reinforced samples were highly effective in keeping a balance between the favorable fibroblast responses and the suppressive microbial growth, which could account for the optimal soft tissue seal in the oral cavity. Furthermore, the underlying mechanism regarding new material’s bactericidal property in the current study has been elucidated as the electron transfer, which disturbed the bacterial respiratory chain and resulted in a decrease of microbial viability. According to the Kyoto Encyclopedia of Genes and Genomes (KEGG) database, the PICRUSt tool was conducted for the prediction of microbial metabolism functions. Consequently, it is inferred that Ti-0.125G has promising potentials for application in implant dentistry, especially in enhancing the integrity of soft tissue and improving its resistance against bacterial infections around oral implants.

## Introduction

The integrity of the soft tissue seal is vital for the long-term success of dental implants, which can prevent bacterial invasion and protect the underneath osseointegration. Nowadays, commercial pure titanium (Cp-Ti) has been commonly used as a transmucosal component of the implant for decades due to its superior biocompatibility, corrosion resistance, and excellent mechanical properties (Yue et al., 2014). However, there are some burning questions demanding prompt solutions, such as Cp-Ti weakly performs in bactericidal effect and soft tissue integration. These limitations make transmucosal applications of implants susceptible to the colonization of oral pathogens, which contribute to the high risks of peri-implant infection and even lead to implant failure (Mellado-Valero et al., 2013).

Graphene (Gr), a two-dimensional carbon material, has not only showed the potential of its excellent biocompatibility to various mammalian cells but also exhibited an ideal bactericidal characteristic to microorganisms (Sanchez et al., 2012; Jia et al., 2016). Currently, Gr is often introduced into titanium by coating techniques such as chemical vapor deposition. However, the benefit of the coating layer is paired with the risk of layer peeling due to the compromised adhesion strength between Gr and titanium surface (Gu et al., 2018). Therefore, in order to tailor Cp-Ti with favorable bactericidal property and fast gingival attachment, a novel Gr-reinforced titanium (Ti-0.125G) was fabricated using the spark plasma sintering (SPS) technique in our study, which made Gr evenly dispersed in titanium with a tight bond.

Various bacterial aggregated and formed as biofilms on the implant surface are often regarded as the prelude of peri-implant infection. Previous investigations mainly used one or two microbes to evaluate the antibacterial activity of biomaterials, which was limited to reflect the reality in nature (Pereira et al., 2015; Schmidt et al., 2016; Sridhar et al., 2016). Here, for evaluating Ti-0.125 G’s antimicrobial property more precisely, we mimicked the oral condition and constructed a multispecies biofilm containing typical pathogens of peri-implantitis like *Streptococci mutans* (*S. mutans*), *Fusobacterium nucleatum* (*F. nucleatum*), and *Porphyromonas gingivalis* (*P. gingivalis*). The dynamic changes of the bacterial multispecies biofilm were subsequently determined by absolute quantification PCR and 16S rRNA sequencing. In addition, although HGFs were mostly selected to assess the soft tissue attachment to biomaterials, the biomaterials’ selective responses to bacteria and HGFs were often neglected. In this regard, a co-culture model involving the aforementioned pathogens and HGFs was established to evaluate the effect of Ti-0.125G on soft tissue seal. This model served as a better mimicry accounting for a “race-for-the-surface” between the bacterial multispecies and HGFs in the transmucosal region (Zhao et al., 2014).

Herein, a novel Ti-0.125G was fabricated by the SPS technique and aimed to be the emergence profile component for implants. The antibacterial activity of the Ti-0.125G against the above multiple pathogens was assessed by morphological observation, live/dead fluorescent staining, spread plate test, and Illumina high-throughput sequencing. Also, the soft tissue responses to Ti-0.125G were evaluated *in vitro*. The selective response of the Gr-reinforced sample was assessed by the co-culture model containing HGFs and the pathogenic multispecies. Besides, an attempt has been made to explain the underlying mechanism of Ti-0.125 G’s bactericidal property. Ti-0.125G has a promising antibacterial potential and it may have a wide range of clinical applications in promoting soft tissue integration (Supplementary Appendix Scheme).

## Materials and Methods

### Sample Preparation

Commercially pure titanium (Cp-Ti) powder (average diameter 30 μm, sphere-like, Grade IV) and graphene powder were obtained from the Institute of Solidification Science and Technology, Shanghai Jiao Tong University. For mixing graphene powder with Ti powder uniformly, we firstly added graphene powder into anhydrous ethanol (Sigma-Aldrich, United States) and performed 2–3 h ultrasonic dispersion. After 1,000–1,500 r/min magnetic stirring and 100–200 r/min ball-milling, the Ti/Gr composite powder (particle size of 10–60 μm) was eventually obtained through 240-mesh sieves. Next, spark plasma sintering (SPS) was carried out in the vacuum environment at 900°C and 50 MPa to obtain disk-shaped samples (10 mm diameter, 1 mm thickness). Then the disks were polished with *SiC* paper (150 rpm, 20 N, EcoMet 30, Buehler, Germany), and washed with anhydrous ethanol, acetone (Sigma-Aldrich, United States), and de-ionized (DI) water sequentially (30 min each) in an ultrasonic bath. The control and the test groups were labeled as Cp-Ti and Ti-0.125G, respectively.

### Characterization of the Sample

The surface morphology of samples was detected using a scanning electron microscopy (SEM; ZEISS Gemini 300). The surface chemical characteristic was roughly detected by analyzing 10 points dispersed on the materials’ surfaces using energy dispersive spectroscopy (EDS; OXFORD Xplore). The points on Cp-Ti were chosen randomly while they were elaborately picked out on Ti-0.125G, including the points inside and outside the graphene-like aggregates (which appeared black under SEM observation), as well as those on the boundary lines (*N* = 10). The phase transformation of the material was detected using X-ray diffraction (XRD; rigaku Ultima IV, Japan) patterns at a step size of 0.02° in a 2θ range of 20-80° (30 kV, 20 mA). The morphology of graphene was semi-quantitatively determined by Raman spectroscopy (λ = 532 nm, LabRAM HR Evolution, Horiba France SAS). The surface hydrophilicity was examined using a water contact angle measurement (DSA100, Kruss). The nano-hardness and elastic modulus of samples were characterized using a nano-indentation test (Agilent Tech, Nano Indenter G200). The surface roughness values involving Ra and Rq were determined using an atomic force microscopy (AFM; Nanonavi E-Sweep). The current-voltage (I-V) characteristics of specimens were obtained using an electrochemical analyzer (Reference 3,000, Gamry) with suitable voltage (−6–6 V) at the frequency of 100 mV/s. The material’s electrochemical characteristics including Mott-Schottky curve were evaluated using an electrochemical system (VersaSTAT 3F, Ametek), and artificial saliva (A7990, Solarbio) was used as the liquid environment for testing.

### Bacterial Multispecies Culture

Some typical oral pathogens, including gram-positive *Streptococcus mutans* (*S. mutans*; UA159), gram-negative *Fusobacterium nucleatum* (*F. nucleatum*; ATCC25586), and *Porphyromonas gingivalis* (*P. gingivalis*; ATCC33277), were cultured in the brain heart infusion (BHI) broth medium (BD) under standard anaerobic conditions (80%N_2_, 10% H_2_, and 10% CO_2_ at 37°C). Pathogens were harvested during the exponential growth phase for further use.

### Bacterial Multispecies Morphology

After incubated with microorganisms for 24 and 48 h, all samples were fixed with 2.5% glutaraldehyde overnight at 4°C. All samples were dehydrated in a series of the ethanol concentration gradient of 30, 50, 75, 90, and 100 v/v% (aq.), and then were freeze-dried, coated with gold, and observed using SEM.

### Live/Dead Staining of Bacterial Multispecies

After incubating for 6 and 24 h, the biofilms on specimens were stained with Live/dead^®^, BacLight^TM^ bacterial viability kit solution (Invitrogen). The 1:1 fluorochrome ratio of 10 min reacting time provided the optimum fluorescence effects. Consequently, the intact bacteria were stained fluorescent green, while the non-viable ones with compromised membranes were stained fluorescent red.

### Spread Plate Test

The spread plate test demonstrated the antibacterial property during a period (6, 12, 24, and 48 h). In detail, 500 μL mixed bacterial suspension (containing ∼10^6^ CFU/mL *S. mutans*, ∼10^7^ CFU/mL *F. nucleatum*, and ∼10^7^ CFU/mL *P. gingivalis*) was added to the glass disk (the blank). Each sample was placed in a 24-well plate to establish the biofilm. Then the adherent bacterial multispecies were collected in an ultrasonic bath (KQ5200E, 200 W, and 50 Hz) after vortexing. After that, 100 μL solution was evenly spread on the sheep blood agar dish with a sterile cell spreader. After being incubated in the anaerobic condition, colonies of multi-bacteria were counted. The relative colony-forming units [RCFU (%)] and the antibacterial rates [Ra (%)] were analyzed by the following equations:

RCFU (%) = (CFU of Cp-Ti or Ti-0.125G)/CFU of the blank × 100%

Ra (%) = (CFU of the control-CFU of the reinforced sample)/CFU of the control × 100%

### Bacterial Biofilm Susceptibility and Viability Assay

Crystal violet (CV) staining and the 3-(4,5-dimethylthiazolyl-2)-2,5-diphenyltetrazolium bromide (MTT) test were used to investigate the bactericidal effect of materials on the formed biofilms. In the beginning, 500 μL BHI medium involving multispecies (∼10^6^ CFU/mL *S. mutans*, ∼10^7^ CFU/mL *F. nucleatum*, and ∼10^7^ CFU/mL *P. gingivalis*) was added on the samples pre-placed in a 24-well plate to develop the biofilms. As for the biofilm susceptibility assay, after incubating for 12, 24, and 48 h, the supernatant was carefully aspirated, and the contaminated samples were rinsed and then fixed with methanol for 15 min at RT. After stained with CV for 10 min, the biofilm-disk complex was washed and dried overnight. The stained biofilm was dissolved with 95% ethanol and its absorbance was recorded at 550 nm wavelength. As for viability assay, after incubating with 0.5 mg/mL MTT for 2 h, the solution was gently decanted before DMSO was added to dissolve the formazan crystals. The absorbance was recorded at 490 nm wavelength.

### Absolute Quantification of Bacterial Multispecies

Absolute quantification PCR (AQ-PCR) was conducted to illustrate the biomasses of bacterial multispecies quantitatively in a well-established biofilm After washed with phosphate-buffered saline (PBS; Sigma) to remove the non-adherent colonies. The biofilms were isolated using a 10-min ultrasonic bath at RT. According to the manufacturer’s instructions, the total DNA extraction was performed using a DNA isolation kit (Omega). After preparing the mixture solution containing DNA, F-/R-primers, and signaling SYBR Green probe (Vazyme, Q112-02), the samples were reacted sequentially under the initial amplification cycle of 95°C for 5 min, followed by 40 cycles at 95°C for 15 s and 60°C for 30 s. Gene expressions were analyzed by corresponding quantification cycle (Cq) values. The primers used in this assay were listed in Table 1 and were commercially synthesized (Personalbio Co., Ltd., Shanghai). The final result (the absolute quantity (X0) of each colony) was obtained according to the following correlation: Cq = -KlogX0 + b. The disposal was repeated in triplicate to ensure repeatability.

**TABLE 1:** The prime sequences of bacteria in absolute quantification PCR.

**Species**	**Sequence (5′ to 3′)**	**Amplicon size (bp)**
*S. mutans*	F: GCCTACAGCTCAGAGATGCTATTC	118
	R: GCCATACACCACTCATGAATTGA	
*F. nucleatum*	F: CAGCTTGCCATACTGCG	404
	R: ACTGTTAGCAACTACCGATGT	
*P. gingivalis*	F: GGCCACAAGGGGACTGAGACA	182
	R: TTTAGCCGTCACTTCTTCTGTTGG	

### Illumina 16S rRNA Sequencing of Bacterial Multispecies

Herein, a 10 mm × 1 mm glass disk was regarded as the blank group due to its insulative characteristic compared to the metal disks, excluding the potential electrochemical interferences. The universal reaction of V3-V4 regions of the bacterial 16S rRNA genes was amplified by 338F/806R primers. PCR was conducted according to the previous instructions (Cheng et al., 2019). The unprocessed data was obtained after the first sequencing with an Illumina Miseq sequencing platform (PE300, Personalbio). After the process of quality control, the resulting sequences were clustered into amplicon sequence variants (ASVs) at a 100% similarity using Quantitative Insights Into Microbial Ecology 2 (QIIME2) dada2 clustering. Diagrams were produced with various aspects based on the abundance of ASVs combined with a set of multivariate analyzing tools. A prediction of microbial metabolism functions was provided using PICRUSt (Phylogenetic Investigation of Communities by Reconstruction of Unobserved States) tools according to the Kyoto Encyclopedia of Genes and Genomes (KEGG) database.

All 16S sequencing data on gene expressions have been deposited in NCBI’s Sequence Read Archive (SRA) and are accessible through accession number PRJNA707007^1^.

### HGFs Culture

The primary HGFs were obtained from the healthy gingival tissue blocks clinically. All protocols related to humans in this study were approved by the Independent Ethics Committee of Shanghai Ninth People’s Hospital affiliated to Shanghai Jiao Tong University School of Medicine (YBKA201906). The gingival tissues were harvested from young adults (18–35 years old, *n* = 5) during oral implant surgeries and cut into fragments with sterile scissors. Then the fragments were covered by glass slices in a 9 cm dish (Corning) and damped with Dulbecco’s modified Eagle medium (DMEM; Sigma) containing 10% fetal bovine serum (FBS; Gibco) and 100 U/mL penicillin/streptomycin and 2 mM glutamine at 37°C in a standard humidified incubator. Primary HGFs were isolated 2 weeks later, and passages 2–7 were used in this study.

### HGFs Morphology on the Sample

For investigating the morphology of adherent cells, SEM analysis was employed. Briefly speaking, HGFs of 1 × 10^4^ cells per well were seeded on the samples on a 24-well plate. After 1 and 6 h of incubation, the samples were transferred to a new plate and fixed in 2.5% glutaraldehyde overnight at 4°C. The samples were dehydrated sequentially using ethanol solution with concentrations of 30, 50, 75, 90, and 100 v/v% (aq.). Then, the samples were placed in hexamethyldisilazane (HMDS) ethanol solutions and freeze-dried overnight. All samples were sputter-coated with gold before further characterization. Measurements were conducted thrice.

Morphologic details of HGFs were also observed using the confocal laser-scanning microscope (CLSM; Leica). The cells (1 × 10^4^ cells/well) were seeded on the disks in a 24-well plate. At 6 and 24 h, the samples were rinsed twice with PBS, followed by fixed with 4% paraformaldehyde (PFA) and permeabilized with 0.1% Triton X-100. Then, the cellular cytoskeletons and nuclei were sequentially stained with phalloidin and DAPI (Invitrogen), respectively. The disposals followed the protocol provided by the manufacture.

### HGFs Adhesion and Proliferation

5 × 10^4^ Fibroblasts per well were cultured with the samples for 1, 4, and 7 days in 24-well plates. After being washed, the specimens were incubated with 500 μL of DMEM plus 50 μL of CCK-8 solution for 1 h at 37°C. The absorbance of the solution was recorded at 490 nm wavelength following the manufacturer’s instructions. Besides, cell nuclei fluorescing with DAPI were detected under CLSM detection, and the disposal followed the aforementioned instructions.

### HGFs Migration

The following experiments detected the migration property of HGFs on different surfaces horizontally and vertically. In wound healing assay, HGFs were grown on the samples overnight with a density of 1 × 10^6^ cells per well until the cells reached confluence. Then, the cell monolayer was wounded carefully with a plastic pipette. After culturing for an additional 0, 6, and 24 h, the cell-disk complex was transferred to another plate and fixed in 4% PFA at each time point. Next, 0.1% Triton X-100 was used to permeate the cell membrane for 10 min. Similarly, DAPI-stained cell nuclei were visualized under CLSM. In fluorescence images, the original wounded region, namely, the area between each border of the monolayer was measured, so as to calculate the number of the migrated cells stained blue in the wounded areas.

In the transwell experiment, 0.4-mm-micropore transwell inserts (Corning Costar, Lowell, MA, United States) were placed upon the samples, which were pre-placed in a 24-well plate. First, HGFs were trypsinized and diluted with serum-free medium. Then, 750 μL DMEM with FBS was added in equal quantity to the lower chamber before 200 μL cell suspension (containing 2 × 10^5^ cells/mL) was pipetted into the inner chamber. The cells were incubated for 12 h and fixed in 4% formaldehyde solution. Before being stained with 0.1 w/v% CV, the cells were permeabilized with 100% methanol at RT. The images of migrated cells were finally recorded under a light microscope (Leica).

### qRT-PCR Analysis of HGFs

The total RNA was extracted using the total RNA isolation kit (Omega), then cDNA was generated using the PrimeScript 1st Strand cDNA synthesis kit (TaKaRa). Analyses were performed using the LightCycler 480 II thermocycler (Roche). The primers in this study were synthesized commercially (Shenggong). The corresponding genes and primer sequences were listed in Table 2.

**TABLE 2:** Primer pairs of HGFs in real-time PCR analysis.

**Gene**	**Primers (F = Forward, R = Reverse)**
VCL	F: CGAATCCCAACCATAAGCAC
	R: GCCGACTTCCTTCACCATAG
ITGB1	F: TGGAGGAAATGGTGTTTGC
	R: CGTTGCTGGCTTCACAAGTA
FAK	F: CTCCTACTGCCAACCTGGAC
	R: GCCGACTTCCTTCACCATAG
FN1	F: GACCGAAATCACAGCCAGTAG
	R: CATCTCCCTCCTCACTCAGC
COL1A1	F: AAGACATCCCACCAATCACC
	R: CGTCATCGCACAACACCTT
GAPDH	F: TGTGTCCGTCGTGGATCTGA
	R: TTGCTGTTGAAGTCGCAGGAG

### Western Blot

HGFs were trypsinized and lysed with a protein extraction reagent. The concentration of protein was recorded sequentially using the Bradford Kit (Beyotime) and the microplate spectrophotometer (Bio-Tek, Epoch 2) at 595 nm wavelength. Proteins (20 μg) were loaded and then electro-transferred to a polyvinylidene difluoride (PVDF) membrane, which was incubated subsequently with specific primary antibodies as previously described (Wang X. et al., 2016). Ultimately, the protein bands were quantified using the imaging system (Vilber, Fusion Pulse 6).

### The Co-culture Model of Bacterial Multispecies and HGFs

Three species (UA159, ATCC33277, and ATCC25586) and HGFs were sequentially seeded on the samples. 25 μL bacterial suspension [containing ∼10^4^ colony-forming units (CFU)/mL *S. mutans*, ∼10^5^CFU/mL *F. nucleatum* and ∼10^5^CFU/mL *P. gingivalis*] was pipetted on the disks in a 24-well plate and cultured anaerobically for 90 min, yielding the volume of 1 × 10^4^CFU/cm^2^ on the specimens (Wang J. et al., 2016). The modified medium (MM) containing ∼10^4^ HGFs was distributed into each well and incubated aerobically for 6 and 24 h at 37°C. Specifically, MM was composed of DMEM with 10% FBS and 2% BHI (Foss et al., 2015), both of which were crucial in a balance without benefiting either the cells or the microbes at significant advantages. Bovine serum was added into MM and functionalized as the crevicular fluid surrounding dental implants. At each time point, HGFs were fixed and stained with phalloidin and DAPI, and their morphologies were visualized using CLSM.

### Statistics

All data were presented as mean ± standard deviation of at least three independent experiments. The data were statistically analyzed by one-way ANOVA. *P* < 0.05 was regarded as significant.

## Results

### Characterization of the Sample

**FIGURE 1:**
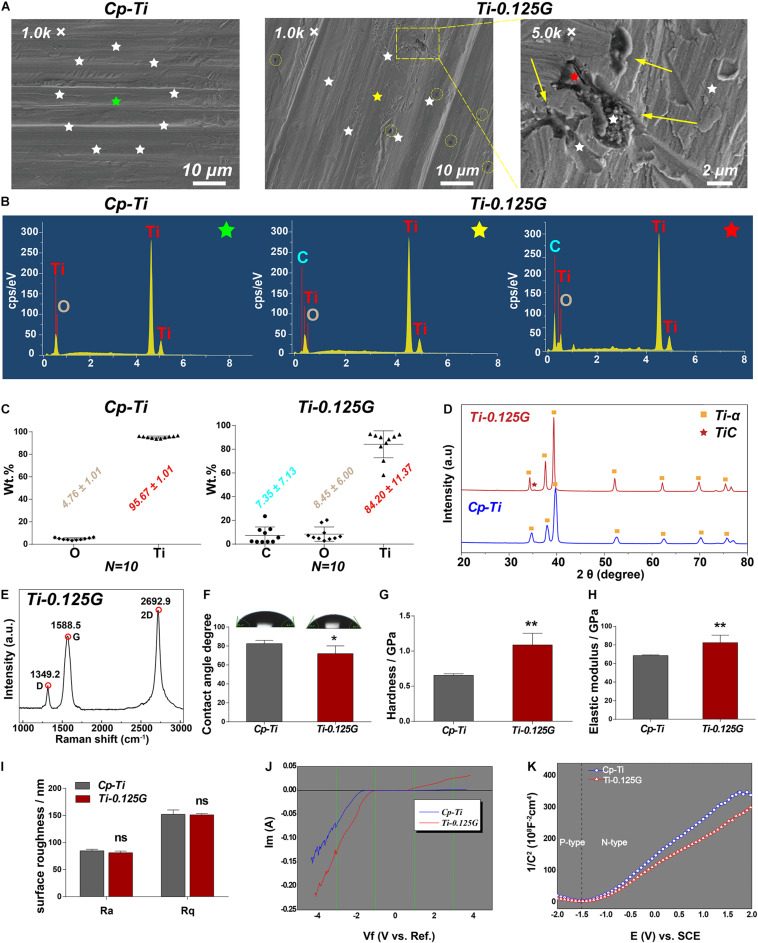
Characterization of the sample. **(A)** SEM micrographs of the samples at different magnification. For the group of Ti-0.125G, the colored circles indicated the distribution of graphene aggregates at 1.0 K × magnification (scale bar = 10 μm), and the arrows marked the agglomerated graphene at 5.0 K × magnification (scale bar = 2 μm). **(B)** The respective EDS points’ spectra of Cp-Ti (green-starred) and Ti-0.125G (the point outside the Gr-like aggregate marked as a yellow star and the point inside the Gr-like aggregate marked as a red star). **(C)** The elemental concentrations of C, Ti, and O were measured by EDS semi-quantitative technique (expressed as average ± standard deviation; *N* = 10). **(D)** XRD patterns of Cp-Ti and Ti-0.125G (2θ = 20–80°). **(E)** Raman spectra of Ti-0.125G. **(F)** Wettability analysis (*p < 0.05). **(G,H)** Elastic modulus and nano-hardness analyses (**p < 0.01). **(I)** Surface roughness. No significant differences in Ra and Rq values. **(J)** The current-voltage (I-V) characteristics. **(K)** The conductivity type was depicted by the Mott-Schottky curves.

We first characterized some key parameters of the Gr-reinforced Ti after the fabrication by the SPS technique. In SEM images, the surface morphology of the two groups seemed to be quite similar except for some black dots existed on Ti-0.125G surface. These dots were like multi-layer graphene aggregates at high magnification (Figure 1A). In contrast to the random emission for the Cp-Ti group, the X-rays for the Ti-0.125G group were designedly emitted to the Gr-like aggregates (black), the surrounding areas (gray) and their boundary lines without overlapping (*N* = 10). The EDS spectra (starred in Figures 1A,B) noted that C peak was unique in the spectrum of the Ti-0.125G compared to the Cp-Ti. And C peak seemed to exhibit a larger and higher shape since the X-ray emission targeted the graphene-like aggregate rather than the surroundings. Also, depending on the 10-point EDS results, we ultimately indicated that the elemental concentrations of the groups were analyzed as O and Ti accounting for 4.76 ± 1.01 Wt.% and 95.67 ± 1.01 Wt.%, respectively, in Cp-Ti and C, O, and Ti accounting for 7.35 ± 7.13 Wt.%, 8.45 ± 6.00 Wt.%, and 84.20 ± 11.37 Wt.%, respectively, in Ti-0.125G (Figure 1C). Figure 1D showed the phase and structure of the Cp-Ti and Ti-0.125G. The XRD patterns of the two groups were similar, and the data were well matched with the International Centre for Diffraction Data (ICDD) reference cards. While Ti-0.125G had a narrow Ti peak at 2θ degree of 39.38 (No. 03-065-9622), which was indexed into the C (101) of multi-layer graphene. Furthermore, the peak intensity of Ti-α/Gr at 34.34°and 37.64° was higher than the pure Ti-α peaks due to the presence of Gr. The peak 2θ = 35.34° (Ti-C peak) indicated graphene existed in the reinforced composites after SPS. Furthermore, the Raman spectrum of Ti-0.125G contained three prominent peaks near 1,349.2, 1,588.5, and 2,692.9 cm^–1^, corresponding to the D, G, and 2D bands of graphene (Fu et al., 2017; Figure 1E).

Moreover, the wettability data showed that water droplets spread more widely on Ti-0.125G with smaller contact angles values compared to Cp-Ti (71.95° ± 3.32° < 82.47° ± 1.48°; *p* < 0.05). The values of Ti-0.125G in elastic modulus and the hardness were significantly higher as compared to the control (*p* < 0.01; Figures 1G,H). The roughness of the two materials showed no significant differences using AFM detections (Figure 1I). In terms of the electrochemical characteristics, when the voltage was -1 volt, the electron flow through both samples ceased (I = 0). As the voltage increased, a thicker oxide membrane such as titanium dioxide was built upon the Cp-Ti to block the electron flow further, which in contrast to the rising current through Ti-0.125G (Figure 1J). The Mott-Schottky curves demonstrated that Ti-0.125G was more electrically-conductive as its carriers were denser than those of Cp-Ti (Figure 1K).

### Bactericidal Ability of the Sample

**FIGURE 2:**
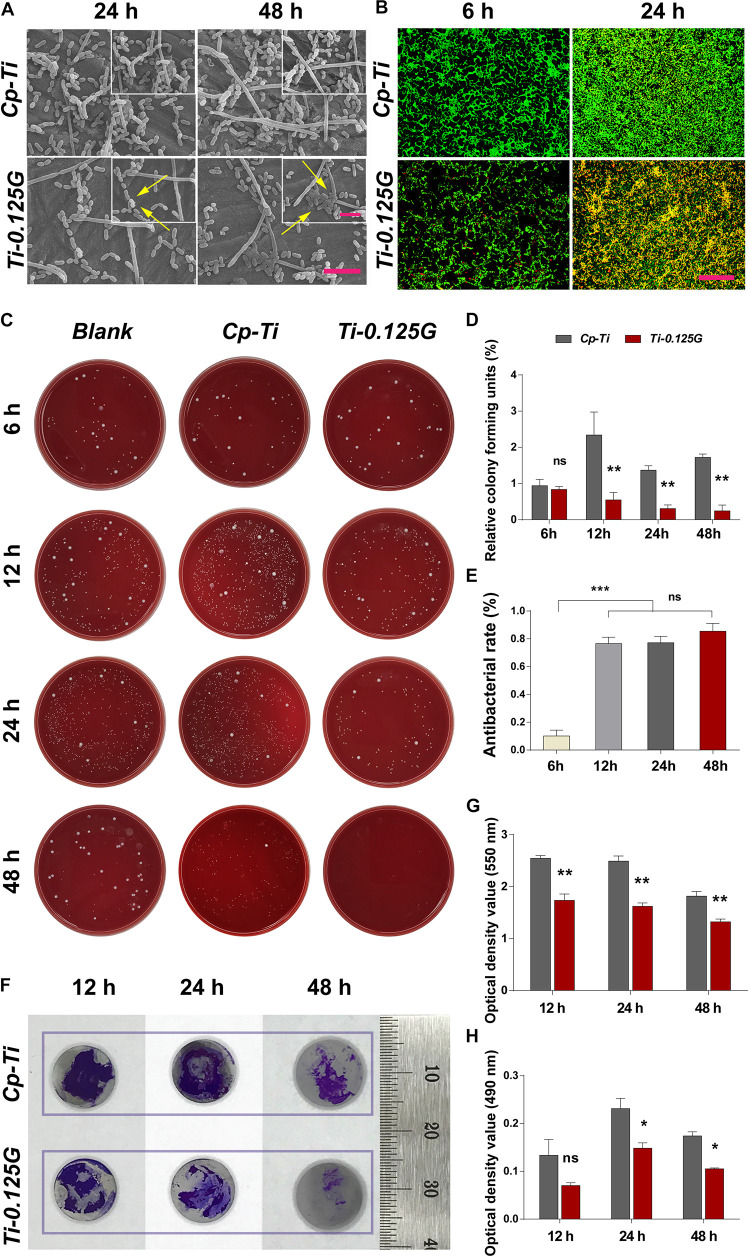
The effects of samples against bacterial multispecies. **(A)** SEM micrographs of multiple bacteria on different samples after 24 and 48 h of incubation. Scale bar = 5 μm. The top right corner insert showed high magnification images (abnormal shapes of microbes were marked by white arrows). Scale bar for inserts = 2 μm. **(B)** CLSM images of bacterial multispecies cultured on different samples for 6 and 24 h. All microbial cells were stained with two well-described probes, interpreting live bacteria (green) and dead ones (red). Scale bar = 50 μm. **(C)** Typical images of re-cultured pathogens (containing *S. mutans, F. nucleatum*, and *P. gingivalis*) colonies on each substrate after 6, 12, 24, and 48 h of incubation. **(D)** and **(E)** Statistical results of relative CFUs and antibacterial rates of all groups. Noted that Ti-0.125G was the most effective in anti-bacteria after 6 h of interaction. **p < 0.01, ***p < 0.001. **(F)** Measurements of biofilm’s biomass by CV staining at 12, 24, and 48 h of incubation. Scale bar was supplemented. **(G)** The statistical analysis noted that Ti-0.125G significantly inhibited bacterial multispecies biofilm. **p < 0.01. **(H)** Statistical analysis of MTT assay noted that Ti-0.125G reduced the viability of bacterial biofilms after 24 and 48 h of incubation. *p < 0.05.

Bacteria of the exponential growth phase were utilized in the study (Supplementary Appendix Figure 1). From a micro-perspective, *S. mutans*, *F. nucleatum*, and *P. gingivalis* exhibited distinct cell shapes on Cp-Ti such as long-chain, long-rod, and short-rod after incubated for 24 and 48 h. Adversely, on the surface of Ti-0.125G, *S. mutans* showed impaired shapes with the flux of intracellular contents, *F. nucleatum* transformed to shorter rod-like structures, and a dozen of *P. gingivalis* remained hardly intact with the collapsed structures (Figure 2A). The fluorescent result depicted that Ti-0.125G decreased multispecies quantitatively compared to Cp-Ti (Figure 2B). From a macro-perspective, the spread plate test indicated that Ti-0.125G exhibited a stronger anti-multispecies potency by largely decreasing the number of CFU from 12–96 h (Figure 2D, *p* < 0.01). Moreover, Ti-0.125G resisted the formation and viability of multispecies biofilm noticeably since it was well-established (Figures 2G,H; He et al., 2017).

### Quantitative and Proportional Analyses of Bacterial Multispecies

**FIGURE 3:**
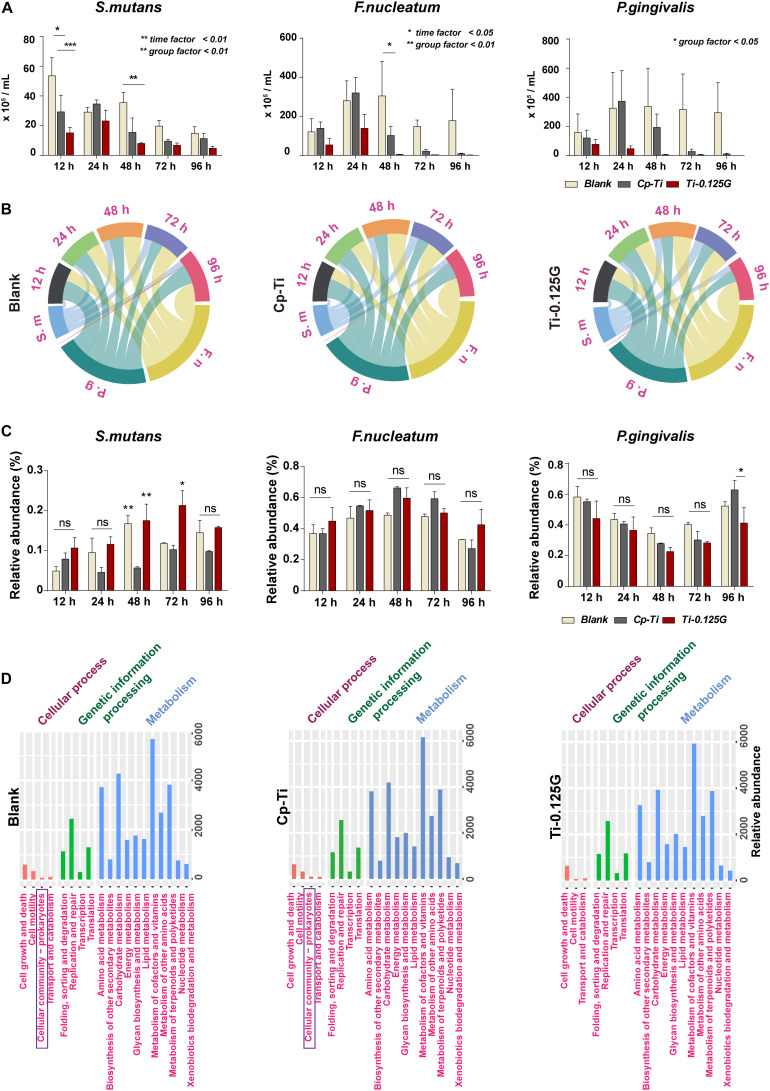
Quantitative and proportional analyses of bacterial multispecies. **(A)** AQ-PCR detected the numbers of pathogens within the multispecies biofilm at 12–96 h of incubation. Dynamic changes in the proportional bacterial biomass in all groups presented in **(B)** a chord diagram and **(C)** a histogram quantitatively. **(D)** PICRUSt2 predictions of microbial functions based on the KEGG database. *p < 0.05, **p < 0.01.

*Streptococci mutans*, the early colonizer of pathogenic multispecies biofilm, decreased substantially in the blank from 12–96 h. *F. nucleatum*, the later colonizers, increased in all groups from 12–24 h and grew to a maximum at 24 h synchronously, then showed a downward tendency in Cp-Ti and Ti-0.125G from 48–96 h (Figure 3A). Reportedly, bacterial multispecies biofilm reached peak viability around implants at 96 h (Bermejo et al., 2019). However, Ti-0.125G highlighted its bactericidal effect with the lowest biomasses of all strains after 96 h of interaction (Table 3). According to the proportional analyses, Ti-0.125G showed the most significant resistance against *P. gingivalis*, a principal etiological agent of implant infection (Zhu et al., 2019), with the thinnest arch in chord diagrams at 96 h (Figure 3B). Additionally, it was indicated that Ti-0.125G did not specifically target a particular strain except inhibiting *P. gingivalis* at 96 h compared to Cp-Ti (Figure 3C and Supplementary Appendix Figure 2). In light of Ti-0.125 G’s favorable bactericidal effect, the mechanism may be relevant to cellular processes based on the KEGG database.

**TABLE 3:** Numbers (expressed as the mean and standard error of the mean) of oral pathogens [×10^5^ colony-forming units (CFU)/mL] detected by absolute quantification PCR throughout the whole culturing duration on different samples, using specific primers and probes aimed to the 16S *rRNA* gene.

**Bacterial species**	**Incubation time (hours)**	***Blank***	***Cp-Ti***	***Ti-0.125G***
		
		**Mean (SEM) (× 10^5^ CFU/mL)**		
*S. mutans*	12 h	53.6 (12.2)	29.3 (11.1)	15.1 (3.7)
	24 h	29.1 (3.2)	34.5 (2.8)	23.2 (7.3)
	48 h	35.4 (6.9)	15.4 (9.7)	8.0 (0.4)
	72 h	19.6 (6.9)	9.5 (1.2)	6.7 (1.6)
	96 h	14.7 (4.5)	11.3 (3.4)	4.7 (1.4)
*F. nucleatum*	12 h	120.9 (67.3)	138.7 (32.5)	53.4 (33.8)
	24 h	279.7 (101.9)	319.8 (80.0)	138.9 (71.3)
	48 h	304.5 (176)	102.3 (46.0)	4.7 (1.0)
	72 h	147.7 (33.3)	21.3 (9.0)	2.6 (1.0)
	96 h	177.7 (161.5)	8.2 (2.8)	1.6 (0.4)
*P. gingivalis*	12 h	157.4 (127.3)	121.3 (52.8)	77.13 (32.47)
	24 h	324.3 (246.0)	373.2 (210.4)	46.76 (17.99)
	48 h	336.2 (262.5)	193.1 (91.2)	4.2 (3.2)
	72 h	315.6 (244.3)	27.1 (16.9)	4.2 (2.9)
	96 h	294.0 (206.0)	10.6 (5.0)	0.4 (0.2)

### HGFs Responses to the Sample

**FIGURE 4:**
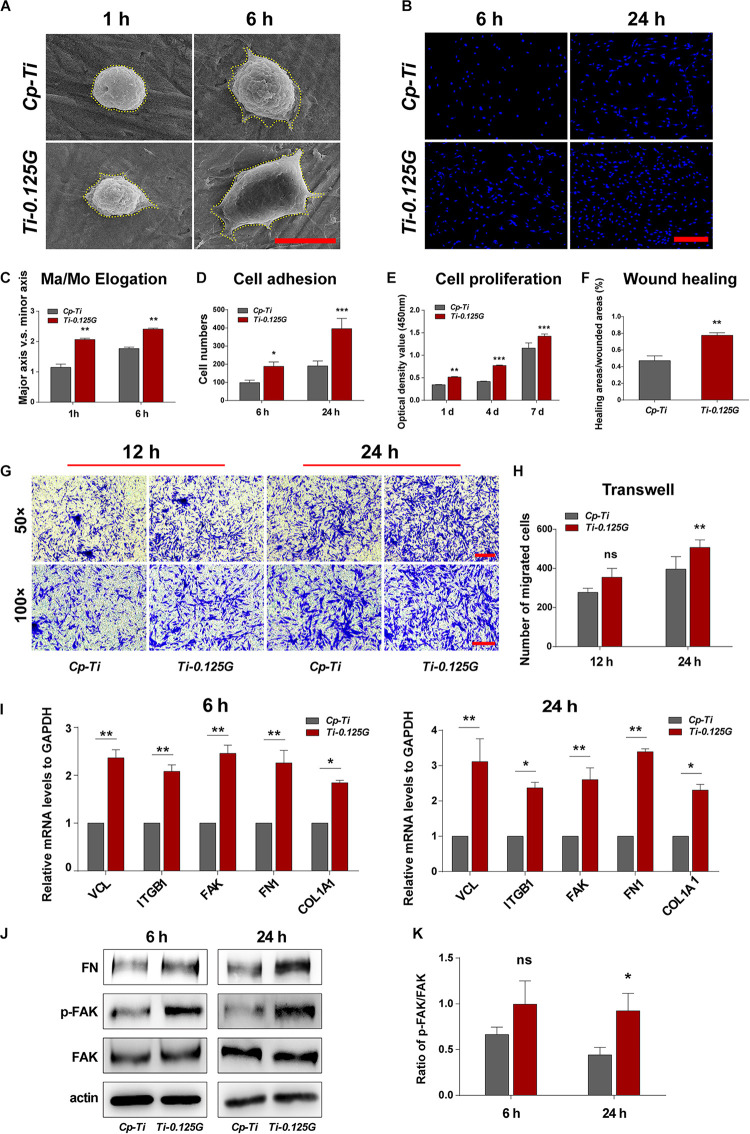
HGFs Responses to the Sample. **(A)** SEM micrographs of HGFs on different samples at 1 and 6 h. Scale bar = 5 μm. **(B)** Initial cell adhesions were observed at 6 and 24 h under CLSM. Scale bar = 250 μm. **(C)** Major axis/Minor axis of HGFs after 1 and 6 h of incubation (**p < 0.01). **(D)** Statistical analysis of cell adhesion at the early stage (*p < 0.05, ***p < 0.001). **(E)** HGFs proliferation properties on different samples were detected *via* CCK-8 for 1, 4, and 7 days (**p < 0.01, ***p < 0.001). **(F)** Statistical analysis of wound healing assay (**p < 0.01) at 24 h. **(G,H)** Transwell assay at 12 and 24 h of incubation, CV-stained HGFs were counted as the migrated cells under the light microscope observation. Scale bar for 50 × /100 × magnification = 200 μm) (**p < 0.01). **(I)** Real-time PCR analyses of cell adhesion- and ECM component-related genes expression (*p < 0.05, **p < 0.01). **(J)** Western blot analyses of signaling proteins, including *FAK*, *p-FAK*, and *FN* **(K)** Increased *FN* adsorption on Ti-0.125G (*p < 0.05).

A series of cellular responses including morphology, adhesion, proliferation, and migration on the sample were investigated in Figure 4. Initially, HGFs exhibited spherical morphologies on Cp-Ti, compared to the varied morphologies with minimal spread on Ti-0.125G. 6 h later, HGFs spread larger with multipolar spindle shapes on Ti-0.125G, resulting in a higher value of Ma/Mo elongation (*p* < 0.01). The similar morphologies were visualized by fluorescent staining on the Gr-reinforced sample (Supplementary Appendix Figure 3). Both at 6 and 24 h, HGFs adhered and increased densely on Ti-0.125G as compared to Cp-Ti (*p* < 0.05). In addition, Ti-0.125G exerted more potential to promote HGFs proliferation by extending the incubation time to 4 and 7 days (Figure 4E, *p* < 0.01). After interacted for 24 h, the horizontal and vertical migration capabilities of HGFs were triggered significantly by Ti-0.125G (Figures 4F,H, *p* < 0.01). Herein, Gr-reinforced sample sparked the HGFs’ responses may be due to the increased expressions of adhesion-related genes (VCL, ITGB1, and FAK) and extracellular matrix (ECM) component-related genes (FN1 and COL1A1) at 6 and 24 h (*p* < 0.05). Moreover, the expression of p-FAK was activated with a higher ratio of p-FAK/FAK on the Gr- reinforced sample (Figure 4K, *p* < 0.05).

### Co-culture Model of Bacterial Multispecies and HGFs

**FIGURE 5:**
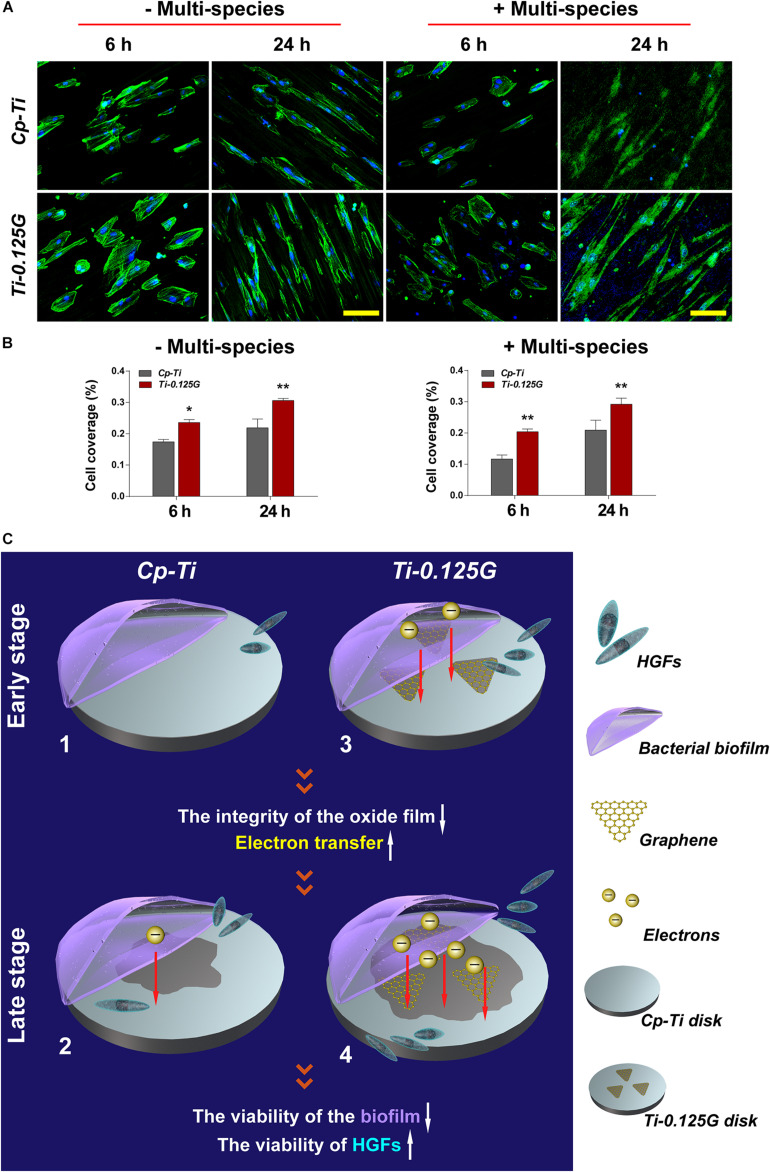
The co-culture model of bacterial multispecies and HGFs. **(A)** CLSM images of HGFs seeded on different samples after 6 and 24 h of contamination by bacterial multispecies biofilms. The green and blue dyes marked the cytoskeleton and nuclei of HGFs, respectively. Scale bar = 100 μm. **(B)** Noted that cell coverage (%) on Ti-0.125G increased as compared to the control (*p < 0.05, **p < 0.01). **(C)** Schematic circuitry of the proposed mechanism to clarify the bactericidal ability of the Gr-reinforced sample. (1) and (3) The primary stages of a “race-for-the-surface” between bacterial multispecies biofilms and HGFs on Cp-Ti and Ti-0.125G, respectively. (2) and (4) The late stages of a “race-for-the-surface” between bacterial multispecies biofilms and HGFs on Cp-Ti and Ti-0.125G, respectively.

Previous studies focused more on a simple cell-bacteria co-culture model consisting of the mono-factor. Currently, we re-designed the study to more closely resemble natural conditions, as showed in Figure 5. In the competition against bacterial multispecies, fewer HGFs covered the Cp-Ti with wizened morphologies, while more robust HGFs grew on the Gr-reinforced sample. In terms of the cell coverage (%), HGFs adhering to the Gr-reinforced surface increased at 24 h (*p* < 0.01), implying that Ti-0.125G had the potential for enhancing soft tissue seal by benefiting the HGFs’ viability in the presence of multispecies.

## Discussion

The current study fabricated a novel composite (Ti-0.125G) for the transmucosal profile and subsequently explored its dual effects regarding antibacteria and fast gingival attachment. Although better fibroblast adhesion is always in contradiction to less bacterial adhesion, the Gr-reinforced sample was expected to manipulate the “race-for-the-surface” between pathogenic multispecies and HGFs, thus to promote soft tissue integration in a pathogen-rich environment within transmucosal regions.

Reportedly, the SPS technique is more accessible to achieve large-scale productions (Rosa et al., 2012) and contributes to a robust bonding of Ti-0.125G, since titanium atoms acts as electron donors and Gr acts as electron acceptors (Subbiah et al., 2014). Chemical vapor deposition (CVD) is the common technology that deposit single-layer graphene sheets on Ti disk. Reportedly, it may face the grim risk of layer peeling due to compromised adhesion strength between Gr and Ti surface (Gu et al., 2018). In contrast, for our material fabricated by SPS technology, which can largely offset the effect of layer peeling due to the strong bonding between graphene and Ti. As the temperature increases, the bonding strength between Gr and titanium increases (Hu et al., 2017). In our study, the temperature elevated above 900°C, Gr reacted with Ti and eventually became TiC, improving the mechanical properties of the composites. Therefore, the elastic modulus and hardness of Ti-0.125G increased compared to Cp-Ti (Figures 1G,H). This advantages Ti-0.125G to be used as transmucosal appliances of the implant as the maximum stress was distributed at implant neck (Alvarez-Arenal et al., 2013), which requires a material with the better mechanical property. Currently, the surface roughness showed no significant variance after mixing with a small dose of Gr (Figure 1I). In terms of the “race-for-the-surface,” HGFs gain more advantages than oral pathogens as bacterial adhesion was inclined to reduce due to the surface roughness of Ti-0.125G was regarded as “minimally rough” (Ra < 0.2 μm) (Albrektsson and Wennerberg, 2004; Jin et al., 2019).

Possessing sufficient antimicrobial property is desirable for an implant component in transmucosal regions. Ti-0.125G was found to destroy the intact structures of *S. mutans*, *F. nucleatum*, and *P. gingivalis* extensively, which refined earlier studies mainly focusing on a single gram-positive or negative pathogen (Akhavan et al., 2011; Zhou and Gao, 2014). Multispecies biofilm was reported to be mature after 96 h of adhering (Bermejo et al., 2019), posing a severe challenge since it compromised the integrity of soft tissue around implants. Herein, we gained a deeper understanding of the bactericidal effect of Ti-0.125G on multispecies biofilms in the first 96 h. Given that the blank group inherently reproduced other biofilm models more than a one-off finding (Sánchez et al., 2011), the reliability of the results was ensured in the study. According to the analyses, Ti-0.125G exhibited an effective bactericidal potency against multiple pathogens broadly without suppressing one strain solely, in addition to its particular inhibitory effect on *P. gingivalis* at 96 h. The PICRUSt2 predictions further deduced that its anti-multispecies property might be related to the vanished pathway of “cell community–prokaryotes” in Ti-0.125G compared to the other groups (Figure 3D), implying that Ti-0.125G largely attenuated the synergistic effects between oral pathogens in multispecies biofilms (Lamont et al., 2002; Periasamy and Kolenbrander, 2009).

Apart from assessing the bactericidal potency, an ideal implant component was also expected to possess excellent bio-affinity and bio-activity. Previously, the Gr-induced biocompatibility was verified roundly in mesenchymal stem cells and periodontal ligament stem cells (Nayak et al., 2011; Xie et al., 2015). In the present study, Ti-0.125G was detected to motivate the viability, adhesion, and proliferation of HGFs *in vitro*, combined with stimulating HGFs’ migration horizontally and vertically (Figure 4), which were crucial for protecting soft tissue integrity. ECM and integrins were also found to regulate soft tissue seal by activating FAK to trigger the downstream signals. Moreover, Ti-0.125G was investigated to activate the vinculin (VCL) and phosphorylate FAK, thus FN was deposited to promote HGFs’ adhesion and mobility subsequently (Culp, 1978; Grinnell et al., 1980). Therefore, Ti-0.125G was recognized to enhance the histological integration of peri-implant soft tissue *in vitro*. Notably, the co-culture model of bacterial multispecies and HGFs was established to provide a more rigorous mimicry of the peri-implant environment. In the present model with multispecies invasion, Ti-0.125G contributed to the intact and distinct morphologies of HGFs (Figure 5A), indicating a stronger antibacterial potency than Cp-Ti. Given the bioactivity aforementioned, Ti-0.125G had the dual effects of serving as a promising transmucosal application by simultaneously benefiting HGFs’ responses and suppressing bacterial growth. This paved the way for preserving a competent soft tissue seal around implants.

Ultimately, an attempt had been made to explore the underlying mechanism of bactericidal property, which could be elucidated as a viewpoint of electron transfer from the bacterial biofilm to the sample (Figure 5C). To the best of our knowledge, once the extracellular region enhanced the uptake of electrons from bacterial membrane respiratory protein, microbes eventually lost their viability as lacking sufficient ATP to supply the respiratory chain (Hartshorne et al., 2009). In the current model, pathogens exhibited negative potentials in artificial saliva (pH = 5.6–7.6) because their isoelectric points (IP) were generally lower than the surrounding pH, which formed the necessary condition for electron transfer. On the other hand, Gr was an ideal conductor and electron acceptor. TiC composite was also reported to facilitate electron transfer (Shen et al., 2015; Fu et al., 2017). Given the addition of Gr, it would be possible to break the integrity of the oxide film on Cp-Ti surface, activating this poorly conductive surface into a metal-semiconductor surface. In this way, Gr activated the sample to receive more electrons by making it more sensitive to conductivity. Also, the Fermi level of Ti-0.125G surface decreased since Gr possessed a bandgap close to 0 eV (Charlier et al., 2007). Hence, the facile transfer of electrons was achieved from the multispecies biofilm to the sample when the bacterial biofilm aligned its Fermi level with that of the Gr-reinforced substrate, which in turn lead to a decrease in microbial viability. Consequently, Ti-0.125G gained stronger resistance against multiple bacteria and won the “race-for-the-surface” to enhance soft tissue seal.

## Conclusion

In conclusion, Cp-Ti and Ti-0.125G were synthesized using the spark plasma sintering (SPS) technique. In the current study, the antibacterial and biological properties of the samples were detected roundly. As compared to the control, Ti-0.125G was stronger to suppress the viability of bacterial multispecies biofilms (including *S. mutans*, *F. nucleatum*, and *P. gingivalis*) and activate HGFs bioactivity. The underlying mechanism of the bactericidal property might be summarized as the electron transfer from the bacterial multispecies biofilms to the Gr-reinforced sample. The 16S sequencing and PICRUSt2 functional predictions provided some instructive results.

## Data Availability Statement

The data presented in the study are deposited in the Sequence Read Archive, accession number PRJNA70700.

## Ethics Statement

The studies involving human participants were reviewed and approved by the Independent Ethics Committee of Shanghai Ninth People’s Hospital affiliated to Shanghai Jiao Tong University School of Medicine No. YBKA201906. The patients/participants provided their written informed consent to participate in this study.

## Author Contributions

HL and JS contributed to the conception, design, data acquisition, analysis, and interpretation, and critically revised the manuscript. JW and SQ contributed to design, data acquisition, analysis, and interpretation, drafted, and critically revised the manuscript. XZ and YL contributed to data acquisition and analysis, critically revised the manuscript. YZ and SW contributed to interpretation, and drafted the manuscript. All authors gave final approval and agreed to be accountable for all aspects of the work.

## Conflict of Interest

The authors declare that the research was conducted in the absence of any commercial or financial relationships that could be construed as a potential conflict of interest.

## Footnotes

1 https://www.ncbi.nlm.nih.gov/sra/
