# Supplementary material for: Graphene-Reinforced Titanium Enhances Soft Tissue Seal
Source: Front Bioeng Biotechnol. 2021 Apr 13;9:665305. doi: 10.3389/fbioe.2021.665305 (PMC8076685; doi:10.3389/fbioe.2021.665305)
Supplement: Supplementary file 1 [file Data_Sheet_1.PDF]

Graphene-reinforced Titanium Enhances Soft Tissue Seal

Jianxu Wei, Shichong Qiao, Xiaomeng Zhang, Yuan Li, Yi Zhang, Shimin Wei, Junyu Shi, and Hongchang Lai

Appendix

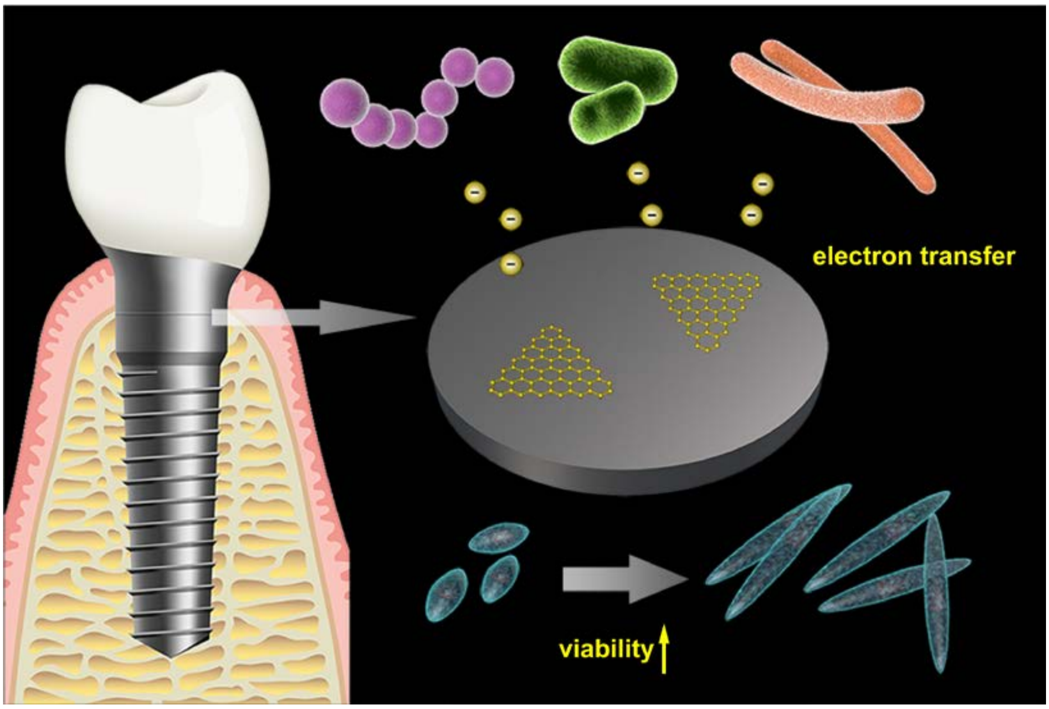

Appendix Scheme. A graphic illustration of Ti-0.125G to keep a balance between the advantageous fibroblast responses and suppressive microbial growth based on the proposed electron transfer theory.

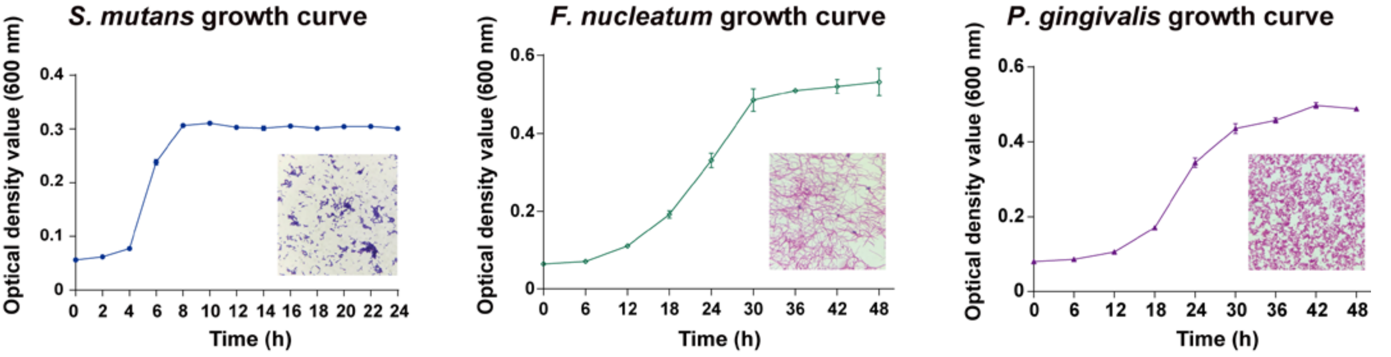

Appendix Figure 1. The typical growth curves of multispecies. The optical values were recorded at a wavelength of 600 nm. The inserts in the bottom right corners were Gram-stained bacteria.

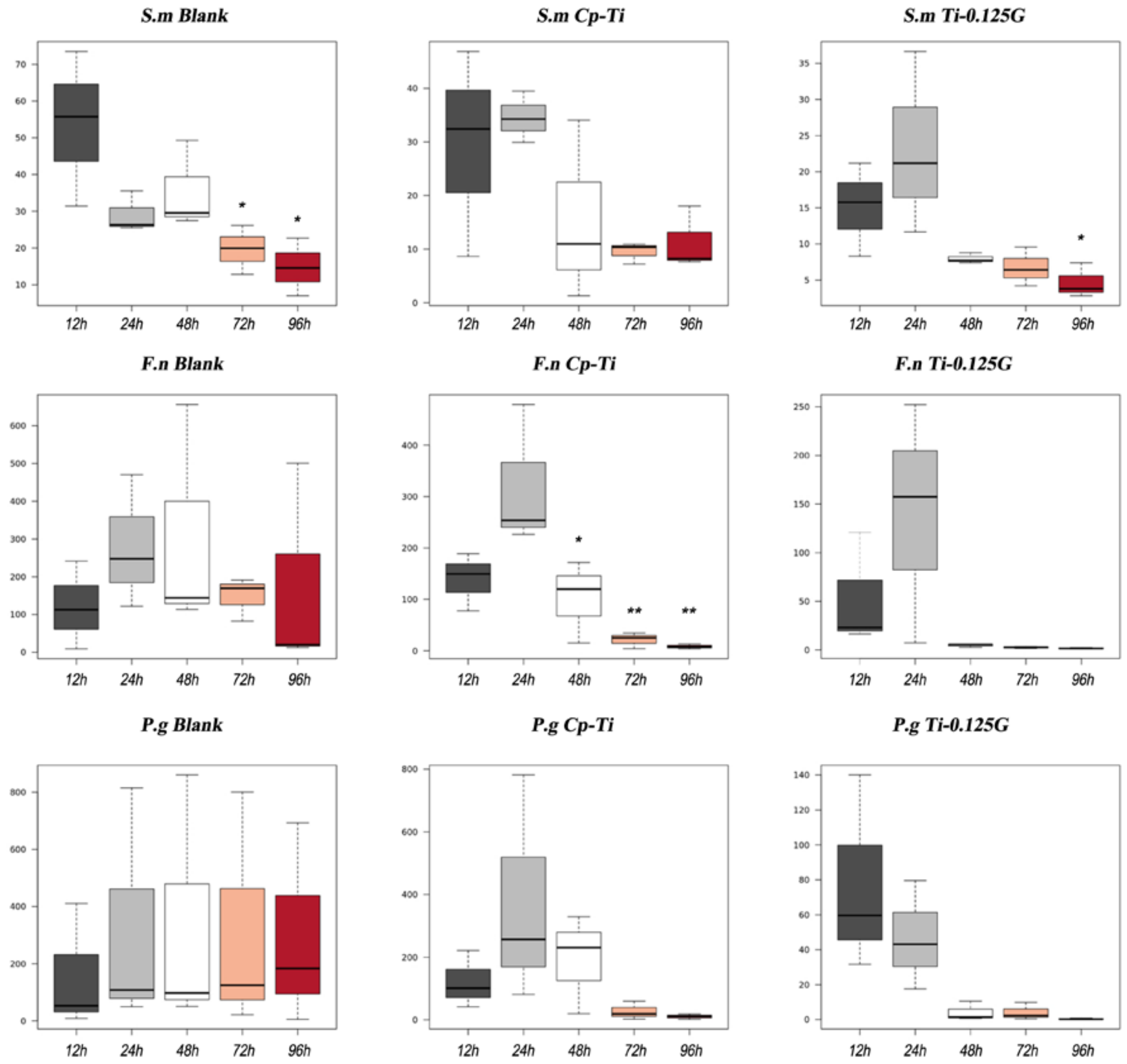

Appendix Figure 2. Box-plot of multispecies determined by AQ-PCR from 12-96 h. *S.m*, *S. mutans*; *F.n*, *F. nucleatum*; *P.g*, *P. gingivalis*. The unit of the horizontal axis was expressed as $\times 10^5$/mL.

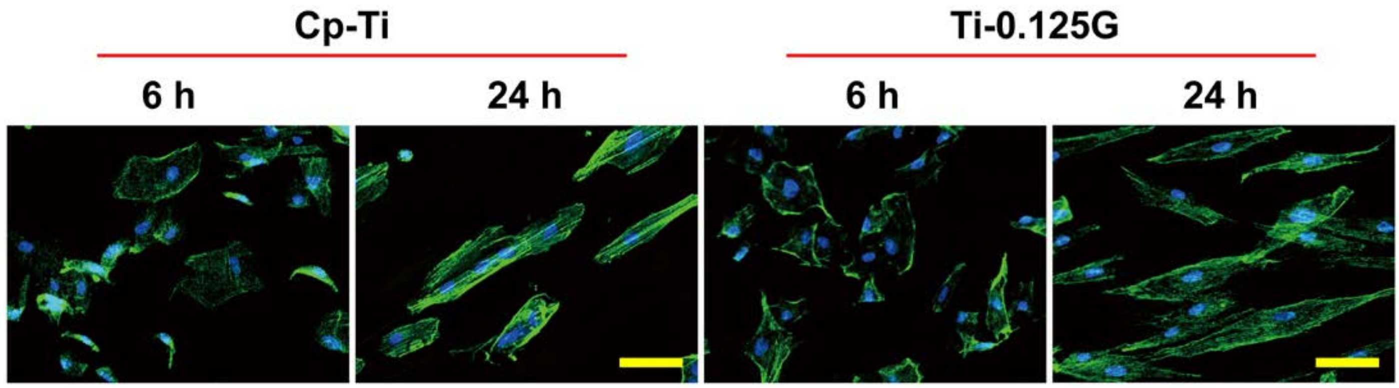

Appendix Figure 3. CLSM images of HGFs stained with DAPI and phalloidin after 6 and 24 h of incubation. Scale bar=50 μm.

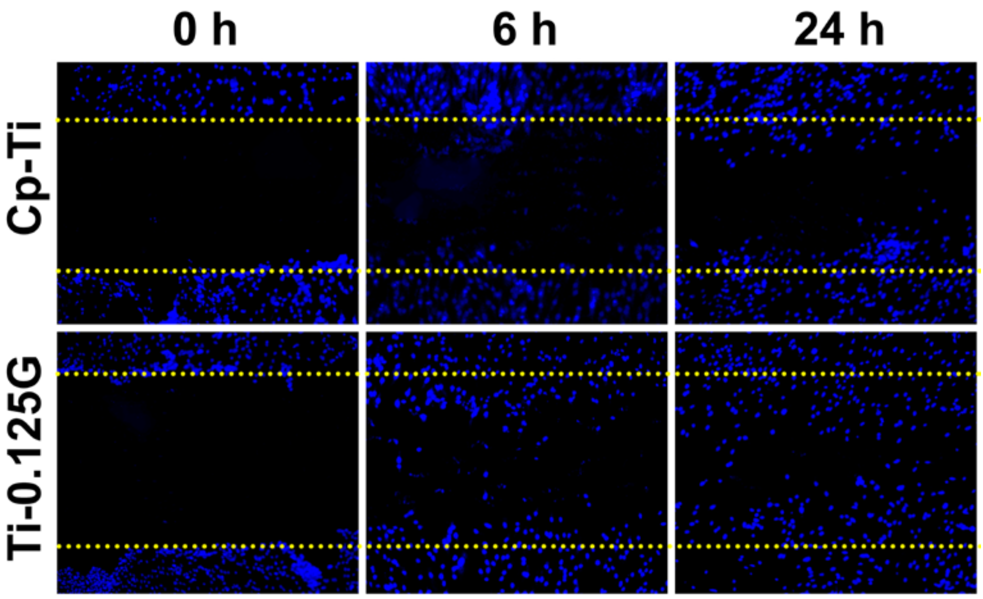

Appendix Figure 4. Wound healing assay of HGFs on different samples. Nuclei (blue) were visualized by DAPI at 0, 6, and 24 h after wounding process. The migrated HGFs covered cell-free areas in a time-dependent manner.
